# A comparison of different linkage statistics in small to moderate sized pedigrees with complex diseases

**DOI:** 10.1186/1756-0500-5-411

**Published:** 2012-08-06

**Authors:** Antònia Flaquer, Konstantin Strauch

**Affiliations:** 1 Institute of Medical Informatics, Biometry and Epidemiology, Chair of Genetic Epidemiology, Ludwig-Maximilians-Universität (LMU) Munich, Neuherberg 85764, Germany; 2 Institute of Genetic Epidemiology, Helmholtz Zentrum München, German Research Center for Environmental Health, Neuherberg 85764, Germany; 3 Institute of Medical Biometry and Epidemiology, Philipps University Marburg, Marburg 35032, Germany

**Keywords:** Linkage, Parametric analysis, Nonparametric analysis, NPL score, LOD score, MOD score, Complex diseases, Rare variants

## Abstract

**Background:**

In the last years GWA studies have successfully identified common SNPs associated with complex diseases. However, most of the variants found this way account for only a small portion of the trait variance. This fact leads researchers to focus on rare-variant mapping with large scale sequencing, which can be facilitated by using linkage information. The question arises why linkage analysis often fails to identify genes when analyzing complex diseases. Using simulations we have investigated the power of parametric and nonparametric linkage statistics (KC-LOD, NPL, LOD and MOD scores), to detect the effect of genes responsible for complex diseases using different pedigree structures.

**Results:**

As expected, a small number of pedigrees with less than three affected individuals has low power to map disease genes with modest effect. Interestingly, the power decreases when unaffected individuals are included in the analysis, irrespective of the true mode of inheritance. Furthermore, we found that the best performing statistic depends not only on the type of pedigrees but also on the true mode of inheritance.

**Conclusions:**

When applied in a sensible way linkage is an appropriate and robust technique to map genes for complex disease. Unlike association analysis, linkage analysis is not hampered by allelic heterogeneity. So, why does linkage analysis often fail with complex diseases? Evidently, when using an insufficient number of small pedigrees, one might miss a true genetic linkage when actually a real effect exists. Furthermore, we show that the test statistic has an important effect on the power to detect linkage as well. Therefore, a linkage analysis might fail if an inadequate test statistic is employed. We provide recommendations regarding the most favorable test statistics, in terms of power, for a given mode of inheritance and type of pedigrees under study, in order to reduce the probability to miss a true linkage.

## Background

Linkage analysis has been a very popular method for detecting genes of major effect. It has been used since the '80s with either sibling pairs or large multiplex pedigrees. In complex diseases, where the mode of inheritance is characterized by factors such as reduced penetrances, presence of phenocopies, and heterogeneity, it has been argued that a better approach to identify variants involved in such diseases is genome wide association analysis (GWAS). In the last years, GWAS have grown in scale and complexity, with studies looking at over a million genetic markers in samples with many thousand individuals. These studies have proved to be successful in identifying common single nucleotide polymorphisms (SNPs) and common risk alleles that contribute to complex diseases. Nevertheless, it is believed that many genetic and epigenetic factors are likely to contribute to common complex diseases, including multiple rare SNPs, i.e., those that occur in less than 5% of the world's population [1]. In fact, it has been argued that these variants are not likely to be captured in current GWA studies due to the low linkage disequilibrium between the rare variant and the more common genotyped SNPs. In such situations a direct mapping approach by means of sequencing techniques will be a useful strategy, although the power to detect single variants by association methods will be low because of their small frequency. Here it will be important to take family information into consideration. Linkage analysis can be a powerful method for detecting the effect of genes for complex diseases [2,3]. Especially because of the enrichment of rare variants in families, linkage analysis has the advantage over association analysis that it is not prone to allelic heterogeneity. That is, the combination of many weak association signals obtained in a certain region for various single variants segregating in different pedigrees is automatically performed in the context of linkage analysis. Therefore, in the era of genome-wide or exome-wide sequencing, linkage analysis has the important task to further restrict the genetic regions that possibly harbor disease causing variants. Anyway, when considering complex diseases, linkage should be used carefully and with some differences compared to Mendelian diseases. It is important to know how the power of linkage analysis is affected by complex inheritance and which sampling schemes and best test statistic should be used to detect disease susceptibility genes in complex genetic diseases.

Genome wide linkage studies are performed with parametric or nonparametric methods. Parametric analysis provides the most powerful method when the mode of inheritance (MOI) is known. The most used parametric statistic is the LOD score [4]. However, because one must assume a MOI for the analysis, the application of parametric methods to complex genetic diseases has been questioned. Therefore, alternative nonparametric linkage methods based on allele sharing by relatives have been developed. Such statistics are the NPL [5] and the KC-LOD score proposed by Kong and Cox [6]. In order to circumvent the difficulty that in parametric analysis the MOI must be specified prior to the analysis, it has been proposed to maximize the maximum LOD with respect to the genetic parameters. This approach is called MOD score analysis [7]. There has been a lot of discussion about the methods that one should use to analyze human linkage data. Also, some research has been done concerning different pedigree sizes. Samples differing in the composition of affected and unaffected siblings in the family will differ in their power to detect linkage when using nonparametric methods [8]. In parametric analysis for a recessive MOI, both linkage and heterogeneity can be detected in feasible sample sizes [9]. For dominant inheritance, linkage can be detected but heterogeneity cannot be detected unless larger sibships (four offspring) are sampled. It has been shown that the distribution of the MOD score is dependent on the size and structure of the pedigrees under study [10]. However, the influence of pedigree structure on the power to detect linkage with the MOD score has not been investigated so far. It is well known that large, multigeneration pedigrees are the most informative for linkage analysis. However, it is not always possible to recruit such families. Given that two-generation families may be the most feasible to study, it is essential to investigate how the sample structure may improve the detection of linkage when complex diseases are present. The present work focuses on the power of parametric and nonparametric linkage statistics to detect the effect of genes for complex diseases using different pedigree structures. We first conducted simulations under the null hypothesis of no linkage to see the influence of pedigree structure on the distribution of the parametric scores (LOD and MOD) and on the nonparametric scores (NPL and KC-LOD). Second, we examine the power of these test statistics to detect linkage for each pedigree structure and discuss which is the best test statistic given the sample under study.

## Methods

### Pedigree structures

**Figure 1:**
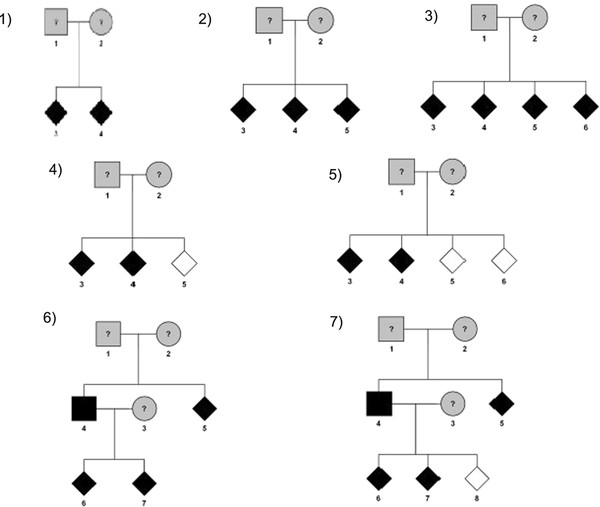
**Pedigree structures used for simulations.** Legend: 1) affected sib pair (ASP), 2) affected sib triplet (AST), 3) affected sib quadruplet (ASQ), 4) discordant sib triplet (DST), 5) discordant sib quadruplet (DSQ), 6) affected three-generation (A3G), 7) discordant three-generation (D3G).

Samples of different pedigree structures and sizes were considered, the same structures as by Mattheisen et al. [10]. Five pedigree structures represent nuclear families, varying the number of affected and unaffected siblings. Furthermore, two structures represent three-generation families (Figure 1). Genotypes are available for all family members. The annotation to each pedigree structure is as follows: affected sib pair (ASP), affected sib triplet (AST), affected sib quadruplet (ASQ), discordant sib triplet (DST), discordant sib quadruplet (DSQ), affected three-generation (A3G) and discordant three-generation (D3G). We conducted also simulations with a mixture of different pedigrees (100 AST, 100 ASQ, 100 DST, 100 DSQ).

### Data simulation

#### Under H_O_

In order to investigate the influence of the pedigree structure on the distribution of parametric and non-parametric linkage scores, 100,000 replicates of 500 pedigrees were generated under *H*_*O*_ using MERLIN software [11] for each of the pedigree structures presented in Figure 1. To allow for a high polymorphism information content, one genetic marker with 8 equifrequent alleles was simulated. Under *H*_*O*_, MERLIN generates each replicate under the assumption of marker segregation independent of the trait, using identical pedigree structure, affection status, marker spacing, allele frequencies and patterns of missing data as given in the input file.

#### Under H_1_

Three scenarios reflecting a dominant, an additive and a recessive MOI were studied to evaluate the power of detecting linkage in complex genetic diseases. For each scenario, 5,000 replicates of 500 pedigrees were generated for each of the pedigree structures shown in Figure 1 under the alternative hypothesis of complete linkage with a dominant (f_0_ = 0.04, f_1_ = 0.20, f_2_ = 0.20, p = 0.05), additive (f_0_ = 0.03, f_1_ = 0.13, f_2_ = 0.23, p = 0.1), and recessive mode (f_0_ = 0.04, f_1_ = 0.04, f_2_ = 0.20, p = 0.2), where f_i_ stands for the penetrance of the genotype with *i* copies of the disease allele and *p* stands for the disease allele frequency. The genetic parameters were obtained using the R package *POWERPKG*[12] for affected sib pairs to obtain a power of approximately 80%. For comparison purposes, these parameters were also used for the other pedigree structures. Heterogeneity was simulated by assuming a nonzero phenocopy rate (f_0_). Phenocopies represent individuals who are affected not owing to genetic predisposition at the locus under study but due to unspecified environmental factors or genes at another location [13]. By the same token, the models include a strongly reduced penetrance. This allows for an individual being unaffected despite having a high risk genotype, either due to environmental or due to other genetic effects. Replicates were generated using MERLIN software [11]. MERLIN simulates data under *H*_*1*_ respecting the pattern and the allele frequencies for the phenotypes and genotypes of the input files, but introduces linkage between the phenotype and the marker depending on the specified MOI.

### Analysis models

To simplify the discussion, we restrict the analysis to a single genetic position. The simulated data were analyzed using parametric and nonparametric methods. The following statistics were used:

#### The parametric LOD

The LOD is computed as the ratio between the likelihood of obtaining the test data if the two loci are indeed linked, versus the likelihood of observing the same data purely by chance. Here, MLINK [14] was used to compute LOD scores letting the recombination fraction vary from 0 to 0.48 in steps of 0.02, and taking the maximum LOD. The program was run using ANALYZE [15]. Three parametric models reflecting realistic scenarios for complex genetic diseases have been specified for the analysis: a dominant model (f_0_ = 0.003, f_1_ = 0.5, f_2_ = 0.5), additive model (f_0_ = 0.003, f_1_ = 0.25, f_2_ = 0.5), and recessive model (f_0_ = 0.003, f_1_ = 0.05, f_2_ = 0.5) where f_i_ stands for the penetrance of the genotype with *i* copies of the disease allele. Since a high disease allele frequency (*p*) can compensate for misspecified penetrance parameters when analyzing complex traits [16], *p* was fixed at 0.25 for all three models. In the following, the term MOI refers to the parametric model used in data simulation and AMOI refers to the parametric model used in the analysis of the simulated data. We have deliberately chosen different parameters in the MOI and in the AMOI, corresponding to the real life situation of studying complex diseases in which the correct parameter values are not known beforehand. Hence, there is some degree of model misspecification even when MOI and AMOI are the same (i.e., both dominant, additive, or recessive). Misspecification of the penetrance does not generally have a strong effect on the two-point LOD score (only on the estimate of the recombination fraction), as long as dominance is specified correctly [7,17].

#### The MOD score

The MOD is based on maximizing the maximum LOD with respect to the trait-model parameters (i.e., penetrance and disease allele frequency). MOD scores were computed with GENEHUNTER-MODSCORE [10,18] with the option “modcalc single”.

#### Nonparametric NPL and KC-LOD score

The NPL focuses on the sharing of disease status as well as the sharing of alleles by relatives. Its extension, KC-LOD, is maximized over a parameter δ representing the degree of allele sharing among affected individuals. Indeed, when the IBD information is complete, there is a one-to-one correspondence between the NPL score and the KC-LOD score, which means that the tests based on the two statistics are formally equivalent [6]. In our case we simulated the models to be as realistic as possible, hence, a highly informative but not fully informative marker was considered. Both statistics were calculated under the “score pairs” option using the MERLIN software [11].

To study the effect of pedigree structure on the distribution of the linkage statistics, each replicate generated under *H*_*0*_ was analyzed using the parametric and nonparametric linkage statistics mentioned above. The scores corresponding to empirical *P-*values of 0.0017 *“suggestive evidence for linkage” *[19], 0.0001 *“classical LOD-3 criterion” *[20] and 0.000049 *“significant evidence for linkage” *[19] were calculated for each statistic and for each structure.

To study the power of the different statistics to detect linkage, each replicate generated under *H*_*1*_ was analyzed using the parametric and nonparametric linkage statistics mentioned above. The values for each statistic were ordered from highest to lowest over the 5,000 replicates for a given model and for a given structure. Observed power levels P(Z) were determined as a function of the score Z used as a critical value for each test statistic T, as follows: P(Z) ≡ (number of replicates yielding T ≥ Z) /N, where N represents the number of replicates performed (i.e., 5,000).

In order to compare the different linkage statistics regarding their power, critical values were obtained using the theoretical distribution under H_0_ for the corresponding test statistic. The NPL score is constructed on the basis of a score statistic [21,22]. Theoretically, once the score statistic is standardized it follows a standard normal distribution. The KC-LOD follows a N(0,1)²/(2·ln(10)) [6]. The LOD score corresponds to a non-standard likelihood ratio statistic, i.e., 2·ln(10)·LOD follows asymptotically half a chi-square distribution with one degree of freedom and half a point mass of zero. The asymptotic null distribution of MOD scores has been derived for ASPs and unilineal affected relative pairs only [23-25], for that reason, critical values for other pedigree structures have to be obtained from simulations under H_0_. Instead of using the critical values obtained in this study, we decided to rely on the ones provided by Mattheisen et al. [10] for the reason that they used one million replicates, i.e., a factor of ten more than we generated in this study, resulting in more precise estimates. In all cases a type I error of α = 0.000049 (significant evidence for linkage) was used. With our three scenarios a considerable number of analyses were required. 4,200,000 analyses were performed under H_0_ ([3 non-parametric scores + 1 parametric score x 3 AMOI] x 7 pedigree structures x 100,000 replicates), and 630,000 analyses were performed under H_1_ ([3 non-parametric scores + 1 parametric score x 3 AMOI] x 3 MOI x 7 pedigree structures x 5,000 replicates).

## Results

### The impact of pedigree structure on the null distribution

**Figure 2:**
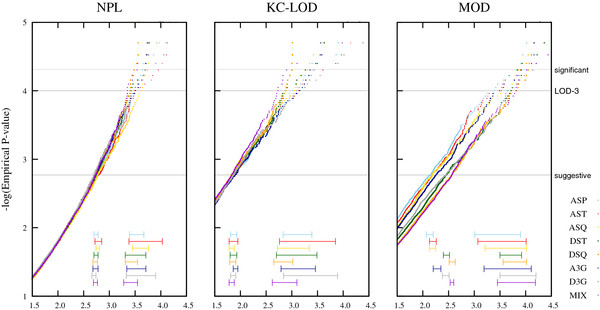
**Influence of the pedigree structure on the distribution of NPL, KC-LOD and MOD score under H**_**0**_**.** Legend: Plots for the empirical distributions regarding the different pedigree sizes (P-values on a logarithmic scale). Horizontal gray lines refer to suggestive evidence for linkage (*P*-value of 0.0017), the classic 'LOD-3-criterion' (*P*-value of 0.0001), and significant evidence for linkage (*P*-value of 0.000049), respectively. The horizontal lines shown at the bottom of each graph represent the 95% confidence interval at the suggestive and LOD-3 level.

**Figure 3:**
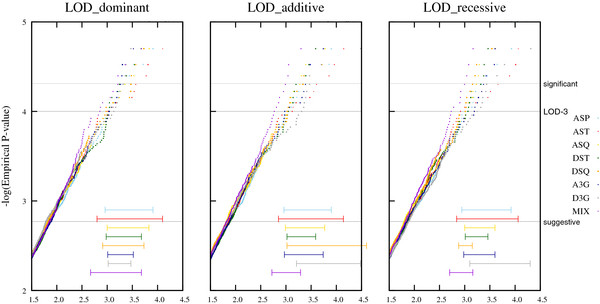
**Influence of the pedigree structure on the distribution of the LOD score under H**_**0**_**.** Legend: Plots for the empirical distributions regarding the different pedigree sizes (P-values on a logarithmic scale) when the replicates are analyzed under the dominant, the additive and the recessive model. Horizontal gray lines refer to suggestive evidence for linkage (*P*-value of 0.0017), the classic 'LOD-3-criterion' (*P*-value of 0.0001), and significant evidence for linkage (*P*-value of 0.000049), respectively. The horizontal lines shown at the bottom of each graph represent the 95 % confidence interval at the LOD-3 level.

A graphical overview of the results is shown in Figure 2 for the nonparametric statistics (NPL, KC-LOD) and the MOD score. The horizontal lines at the bottom of the graph represent the 95% confidence interval at the suggestive and LOD-3 level. The parametric LOD score is shown in Figure 3, with the 95% confidence intervals at the LOD-3 level. Two non-overlapping intervals for two pedigree structures at the same level represent statistically significant differences for the test statistic. Although an effect of the pedigree structure on the distributions of the NPL and KC-LOD score can be appreciated at the LOD-3 level, this effect is not statistically significant. However, there is a significant effect of the pedigree structure on the distribution of the MOD score at the suggestive level (Figure 2). In summary, adding one affected offspring to the ASPs (ASTs, red line) leads to an increase of the MOD score and adding another affected offspring (ASQ, yellow line) leads to a further increase (3.39 vs. 3.53 vs. 3.72; c.f. Figure 2). These results corroborate the findings by Mattheisen et al. [10]. We also see an increase of type I error when analyzing a mixture of pedigrees corresponding to a very similar critical value as DSQ pedigrees (3.81, 3.82 respectively, violet and orange lines). Although the differences among ASP, AST and ASQ are not significant, this group shows significant differences compared to DST and DSQ, i.e., when adding one or two unaffected siblings to ASPs. Significant differences are also found between the three-generation pedigrees (A3G and D3G).

### The power of the test statistics to detect linkage for different pedigree structures

The observed power of NPL, KC-LOD, MOD and LOD scores for each pedigree structure is shown in Additional file 1: Table S1. It compares the power for each pedigree structure (columns) to reach or exceed a given Z value for each test statistic (rows) using a type I error of α = 0.000049 (significant evidence for linkage). The power is given when analyzing the replicates generated under a dominant, additive and recessive mode of inheritance (MOI). In the case of parametric statistics AMOI refers to the mode of inheritance used to analyze the simulated data. Colored numbers are used in the conclusion section for a better understanding of the results. The results for the different statistics are described in the following.

#### Nonparametric NPL

The NPL score is computed using affected pairs only. For that reason, very similar results are expected for ASP, DST and DSQ as well as for A3G and D3G. In fact, the minor differences arise because the disease locus genotypes are conditioned on the phenotypes of the ascertained individuals. A very poor power (8.4%-42.0%) is obtained with ASP, DST or DSQ. The power is increased (99.5%-99.9%) when adding an affected sib (AST), and it is even better (99.6%-100%) when A3G and D3G structures are used. The best power is obtained using ASQ (100%). At the level of significant evidence for linkage, a bad performance of the NPL is acquired when using pedigrees with less than three affected sibs, especially when the MOI is recessive.

#### Nonparametric KC-LOD

The KC-LOD is also computed using affecteds only. For that reason, very similar results are observed for ASP, DST and DSQ as well as for A3G and D3G. An effect of pedigree structure is also obtained with the same trend as for the NPL score, although at the level of significant evidence for linkage, a better power is obtained with the KC-LOD than for the NPL statistic (see Additional file 1: Table S1).

#### MOD

The effect of the pedigree structure on the power to detect linkage using the MOD score depends on the true MOI. When considering a complex genetic disease with a true dominant or additive MOI, ASQ, A3G and D3G provide the best performance. Generally, for all pedigree structures the power seems to be better when the true MOI is dominant. Considering a recessive MOI, again the ASQ and A3G structures provide the best performance in detecting linkage. The MOD score slightly decreases when adding one or two unaffected sibs to the ASP structure, i.e., for DST and DSQ. However, due to the markedly increased critical values for significance (4.20 and 4.36 for DST and DSQ, respectively, compared to only 3.61 for ASP), the MOD score provides a less powerful test with DST and DSQ than with ASP.

#### Parametric LOD

When the true MOI is dominant and the AMOI is also dominant, then AST, ASQ, A3G and D3G structures are performing well. Surprisingly, the power slightly increases when the AMOI is misspecified as being additive. When the true MOI is additive then the additive AMOI performs best, as one would expect, with the dominant or recessive AMOI having only slightly decreased power. Similarly, for a recessive MOI, the best power is obtained if the AMOI is recessive as well. Overall, when the true MOI is recessive the power ranges are much lower than if a dominant or additive MOI underlies the trait. At the level of significant evidence for linkage a very similar power is obtained as the one obtained using the KC-LOD score.

As expected, the results of our simulations show that the power to detect linkage is low, at least with samples of 500 pedigrees, when using ASP, DST or DSQ to map complex diseases. Considering a type I error of 0.000049 ( Additional file 1: Table S1), the lowest power is achieved when the true MOI is recessive, in this case the power to detect linkage with this kind of pedigree structures ranges from only 8.4% (NPL, DSQ) to 20.2% (MOD, ASP). For a non-recessive MOI, 53.4% is the maximum power obtained with these structures (KC-LOD and LOD). The power decreases every time that an unaffected sib is added to the pedigree structure. For example, considering the NPL score with a dominant MOI, the power for ASP is 42%, when adding one unaffected sib it decreases to 36.7% (DST), and with another unaffected sib it decreases to 33.4% (DSQ). This tendency is observed for all the linkage statistics considered. An improvement is achieved when using A3G and D3G, displaying a power from 84.1% to 100%. With such structures the lowest power is obtained when the MOI is recessive. Evidently, the best power is reached when considering the larger pedigree structures, AST (99.5%-100%) and ASQ (100%). In other words, with AST and ASQ, chances are high to map the disease-causing gene, at least for the genetic effect sizes and the MOI simulated here.

With LOD-score analysis, the additive AMOI outperforms the dominant AMOI when the true MOI is dominant. This counterintuitive observation is caused by the fact that the penetrances of the AMOI have been defined to differ from those of the corresponding MOI. It reflects the real-life situation of complex trait mapping in which the true disease model is unknown. This effect can be explained by looking at the points in the triangular parameter space for ASPs that correspond to the true models as well as those specified for the analysis. It turns out that with reduced penetrance and phenocopy rates that we have specified here, the range of parameters within the possible triangle covered by a LOD score analysis under the additive AMOI comes much closer to the point corresponding to the true dominant MOI than the parameter curve induced by the dominant AMOI does (data not shown).

## Discussion

We undertook this simulation analysis to answer two questions. First, what is the impact of the pedigree structure on the null distribution of NPL, KC-LOD, LOD and MOD score statistics? Second, what is the power of the NPL, KC-LOD, LOD and MOD score statistics when a complex disease is present? Results show that an effect of the pedigree structure under no linkage is seen only on the MOD score. The critical MOD score values for suggestive evidence for linkage are markedly increased for DST and DSQ compared to ASP (green and orange lines are much lower than the cyan line in Figure 2). The null distribution of the MOD score has been derived theoretically for ASPs [23] and for unilineal affected relative pairs [25]. By the equivalence between the possible triangle test [26] and MOD score analysis, Knapp et al. [23] found a mixture of chi-square distributions with 2, 1 and 0 degrees of freedom for ASPs. Because the pedigree structure has an important effect on the distribution of the MOD score it would not be appropriate to generally approximate the null distribution by a chi-square distribution with fixed degrees of freedom. Hence, it is impossible to use a Z threshold common for all situations, by now the best way is via simulations.

Regarding our second question, results show that the statistic used plays an important role in the power to detect linkage. In general, all the statistics perform very poorly when ASP, DST or DSQ are considered. This is not surprising given the smaller number of meioses. Assuming a type I error of 0.000049, with such pedigrees the weakest statistic is NPL showing a power of at most 42%, followed by the MOD with power of at most 48.5%, and finally a very similar power is achieved by LOD and KC-LOD statistics with a highest power of 53.4%. A considerable improvement in power is acquired when AST, ASQ, A3G and D3G are used. In general, the lowest power is achieved when a disease is inherited in a recessive pattern.

Clearly, it can be expected that larger pedigrees provide more linkage information than small ones. This is in fact what we principally have observed in our simulation study. Nevertheless, a decrease of power is observed in all statistics when adding one or two unaffected sibs to ASP, resulting in DST and DSQ, respectively. For the nonparametric statistics (NPL and KC-LOD), this can be explained by the fact that only the affected phenotype is used to calculate the statistic, and there are on average less mutant alleles segregating in DST or DSQ than in ASP because the disease-locus genotypes are conditioned on the trait phenotypes of the ascertained individuals. In the case of the parametric LOD score which makes use of both, the affected and unaffected phenotype, this finding is less evident. It could be explained by the fact that trait-model parameters need to be specified prior to the analysis and a misspecification of these parameters has a more aggravating effect with larger sibships [7]. Still the same trend of decreasing power is observed with MOD score analysis which includes a maximization over trait-model parameters, so that the model misspecification is not an issue. However, the critical values for significance are markedly increased for DST and DSQ compared to ASP (4.20, 4.36 and 3.61, respectively), resulting in a MOD score analysis with DST or DSQ being less powerful than with ASP. In fact, in MOD score analysis, effectively a larger number of dimensions of the parameter space is explored in the maximization when analyzing larger pedigrees, which corresponds to a larger number of degrees of freedom regarding the underlying distribution under the null hypothesis of no linkage [25]. It is only worthwhile to 'pay' the associated price of an inflated critical threshold for declaring linkage if at least some degree of information regarding the additionally modeled parameter is in fact present in the data. In our case, we have observed that modeling unaffected individuals is not worth the price of an increased critical value, since due to the strongly reduced penetrance used for simulating the data (at most f_2_ = 0.23), unaffected sibs carry only very limited information with regard to their unobserved trait-locus genotype. The aforementioned explanation particularly applies to a MOD score analysis, but the finding that including unaffected individuals leads to a reduced power to detect linkage in the case of reduced penetrance holds for all parametric and nonparametric linkage statistics investigated in this simulation study. We want to emphasize that simply not genotyping an unaffected sibbling will not correct this deficiency, as it is an issue with the type of pedigree from which siblings are ascertained rather than the analysis method or who in the family is genotyped.

The question arises which test statistic should be used for a given type of sample, in order to obtain a power as high as possible. Additional file 2: Table S2 shows which statistic(s) perform best given the sample structure. As can be seen from Additional file 1: Table S1, this decision depends not only on the type of pedigrees but also on the true MOI. Each color in Additional file 1: Table S1 and Additional file 2: Table S2 correspond to a specific test statistic. If the sample under study consists of ASP or AST then the best statistics are the KC-LOD (green) and LOD (yellow) when the MOI is dominant or additive, but more powerful is the MOD (blue) when the MOI is recessive. In the case of DST or DSQ, under a dominant or additive MOI we would recommend to use the LOD (yellow) under an additive AMOI or the KC-LOD (green), and the LOD (yellow) with recessive AMOI when the true MOI is recessive. When A3G or D3G are analyzed and the MOI is recessive, the best power is achieved with the LOD (yellow) under a recessive AMOI or with the MOD score (blue).

We have to notice that all our results are based on a highly informative marker. It is expected that with a reduced information content the power would decrease. Indeed, there are other differences between real data sets and our simulation settings. For example, in most cases data sets consist of pedigrees of different structure. To give an idea regarding the power in this situation, we conducted the analysis with a mixture of pedigrees (100 AST, 100 ASQ, 100 DST, 100 DSQ) (last column from Additional file 1: Table S1). For this scheme of pedigrees we found a power ranging from 75.7% to 98.7%. Interestingly, in this configuration the MOD score is the most powerful test statistic (despite a high critical value of 4.01 obtained from the simulations under H_0_ performed in this study). Apparently, in the case of a mixture of pedigrees with varying numbers of affected and unaffected children, it is worthwhile to jointly explore all dimensions of the parameter space corresponding to a MOD score analysis with each of the different pedigree types, such that the higher score outweighs the increased critical value that is required to declare linkage. Anyway, this result applies only to the specific mix sample considered in this analysis; if the mix sample changes the power values will also change. Another important difference compared to real data sets is that large pedigrees containing multiple affected members are usually rare for complex traits, especially those with late onset. This is due to relatively small recurrence risks for complex diseases. Small pedigrees, such as nuclear families, e.g. ASP, are relatively common and easier to collect. In these situations, an alternative would be to increment the number of pedigrees to be analyzed. To give at least a hint regarding the number of ASP needed to achieve the same power as with AST, we did simulations for the KC-LOD and obtained that one would need approximately 1500 ASP to reach the same power as with 500 AST.

We chose to use two-point analysis (i.e., single marker linkage analysis) rather than multipoint analysis, because the former requires significantly less computation time, which is especially important in a large-scale simulation analysis. Furthermore, the conclusions for multipoint analysis do not seem to differ substantially from those for two-point analysis [27]. In fact, similar results for two-point and multipoint analysis can be expected for the nonparametric statistics and the MOD score. However, in two-point parametric analysis, the LOD score is maximized with respect to the recombination fraction as we have done here. In the multipoint situation, such a free maximization does not take place. Rather, when the disease-locus position is varied in a multipoint analysis, it is confined to lie between flanking markers, which does not happen in the two-point situation. For this reason, multipoint LOD score analysis is particularly sensitive to a misspecification of the trait-model parameters, such that assuming a wrong trait model may even lead to the exclusion of linkage [17]. Furthermore, it has been shown that multipoint LOD scores no longer follow a chi-square distribution [28]. Multipoint LOD score analysis should therefore be used with caution when complex traits are under study.

Another concern in this study could be that the decreasing cost of SNP genotyping has made it much harder to justify the use of microsatellite genotyping in linkage studies. SNPs are likely to be the genotyping method applied to samples currently being collected. Still, our results should also be valid in the presence of SNP data. In a study of linkage analysis with imprinting, ASP and ASQ were simulated. The study considered first 40 SNPs (MAF 0.15 and 0.32 cM apart to each other) and then one microsatellite marker (with 4 alleles). Results showed no substantial difference in the power to detect linkage between the analysis with SNPs and the analysis with the microsatellite (manuscript in preparation). When using SNPs, one should only be cautious about the closely spaced SNP markers and to model LD, or exclude SNPs in LD before using linkage analysis.

Generally it is difficult to realistically simulate the MOI of complex diseases. Here we have focused on three realistic models with reduced penetrance as well as phenocopies, allowing for other genetic and/or environmental factors. We believe that these set of model parameters, together with their moderate genotype-relative risks, reflect a realistic scenario.

## Conclusion

Linkage analysis is not hampered by allelic heterogeneity, and so it genuinely achieves the combination of the peak signals for many single variants obtained in a certain genetic region. We have demonstrated how important is the impact of pedigree structure on the power to detect linkage when diseases with a complex MOI are considered. The pedigree structure has a more severe effect than a model misspecification in the case of two-point parametric LOD score analysis. Based on these results, it is important to use moderate to large pedigree structures with at least three affected members, unless a very large number of ASPs is available for study, and to be cautious when modeling unaffected individuals with complex diseases. When using small pedigrees, one might miss the true genetic linkage when actually a real effect of linkage exists. We believe that missing a true location is an error at least as severe as the false conclusion of linkage. Furthermore, we have shown that the test statistic has an important effect on the power to detect linkage. Here, according to our simulation results we have provided guidelines to researchers regarding which test statistic should be used when studying a certain pedigree structure. By this means, it is ensured that the collected pedigree data are exploited in the best possible way. After all, such extensive gene-mapping studies should not fail to detect linkage due to a suboptimal test statistic used for the analysis.

## Competing interests

The authors declare that they have no competing interests.

## Authors’ contributions

AF made substantial contributions to conception and design, carried out all the analyses and interpretation of data. KS made substantial contributions to conception and design, revised the manuscript critically for important intellectual content; and gave final approval of the version to be published. Both authors read and approved the final manuscript.

## Supplementary Material

Additional file 1 **Table S1.** Power (%) to achieve a given Z value or higher, for each statistic and for each pedigree structure. Click here for file

Additional file 2 **Table S2.** Statistics to be used given the sample structure. Click here for file
