# Supplementary material for: A comparison of different linkage statistics in small to moderate sized pedigrees with complex diseases
Source: BMC Res Notes. 2012 Aug 6;5:411. doi: 10.1186/1756-0500-5-411 (PMC3475142; doi:10.1186/1756-0500-5-411)
Supplement: Additional file 1 — Table S1. Power (%) to achieve a given Z value or higher, for each statistic and for each pedigree structure. [file 1756-0500-5-411-S1.doc]

***Table 1.*** Power (%) to achieve a given Z value or higher, for each statistic and for each pedigree structure.

|  | |  | **PEDIGREE STRUCTURE** | | | | | | | |
| --- | --- | --- | --- | --- | --- | --- | --- | --- | --- | --- |
| **STATISTIC** | | **MOI** | **ASP** | **AST** | **ASQ** | **DST** | **DSQ** | **A3G** | **D3G** | **MIX** |
| **NPL (Z=3.895491)** | |  |  |  |  |  |  |  |  |  |
|  | | Dom  Add  Rec | 42.0  40.0  11.6 | 99.9  99.6  99.5 | **100**  **100**  **100** | 36.7  36.9  09.2 | 33.4  37.3  08.4 | 100  100  99.9 | 100  100  99.6 | 96.6  85.5  84.6 |
| **KC-LOD (Z=3.295177)** | |  |  |  |  |  |  |  |  |  |
|  | | Dom  Add  Rec | **53.4**  **51.5**  15.5 | **100**  **99.8**  99.6 | **100**  **100**  **100** | **48.1**  **49.1**  11.8 | **44.9**  **49.3**  11.5 | 100  100  99.9 | 100  100  99.8 | 97.2  88.0  82.8 |
| **MOD (Z by simulations)** | | | (3.61) | (3.68) | (4.08) | (4.20) | (4.36) | (4.01) | (4.30) | (4.01*^a^*) |
|  | Dom  Add  Rec | | 48.5  46.6  **20.2** | 99.9  99.6  **99.8** | **100**  **100**  **100** | 37.0  35.9  13.1 | 33.7  34.4  10.2 | 100  100  **100** | 100  100  **99.9** | **98.7**  **90.4**  **95.3** |
| **LOD (Z=3.295177)**  **AMOI: Dom** | Dom  Add  Rec | | **53.1**  **51.1**  17.1 | **100**  **99.7**  99.7 | **100**  **100**  **100** | 44.2  41.2  10.7 | 39.7  36.4  08.7 | 100  100  95.4 | 100  100  84.1 | 93.9  80.4  75.7 |
| **AMOI: Add** | Dom  Add  Rec | | **53.4**  **51.4**  15.4 | **100**  **99.8**  99.6 | **100**  **100**  **100** | **50.1**  **48.0**  11.7 | **48.4**  **47.0**  11.1 | 100  100  99.5 | 100  100  99.5 | 97.9  89.1  85.1 |
| **AMOI: Rec** | Dom  Add  Rec | | **53.2**  **51.4**  17.6 | **100**  **99.8**  99.7 | **100**  **100**  **100** | 49.5  47.3  **13.5** | 47.4  46.0  **12.6** | 99.9  99.5  **100** | 99.6  99.0  **100** | 98.2  89.7  89.2 |

Legend

MOI: Mode of inheritance used in data simulation. AMOI: mode of inheritance used to analyze the simulated data with the parametric statistics. Critical Z values are obtained using the theoretical distribution under H_0_ of the corresponding test statistic and using a type I error of α=0.000049 (level of significant evidence for linkage).The theoretical distributions are:

Critical Z values for MOD score (in parenthesis) are obtained by simulations under H_0_ for each pedigree structure [12].

*^a^* Z value is obtained by simulations under H_0_ based on this study.

Colored numbers are mentioned in the discussion section and also represented in Additional file 2: Table S2 for a better understanding of the results.
