# Supplementary material for: A comparison of different linkage statistics in small to moderate sized pedigrees with complex diseases
Source: BMC Res Notes. 2012 Aug 6;5:411. doi: 10.1186/1756-0500-5-411 (PMC3475142; doi:10.1186/1756-0500-5-411)
Supplement: Additional file 2 — Table S2. Statistics to be used given the sample structure. [file 1756-0500-5-411-S2.doc]

**Table 2. Statistics to be used given the sample structure.**

| **Sample structure** | **Best Statistic** |
| --- | --- |
| ASP or AST | KC-LOD/LOD_ALL_(D,A), MOD(R) |
| DST or DSQ | KC-LOD/LOD_A_(D,A), LOD_R_(R) |
| D3G | LOD_R_(R), MOD(R) |
| ASQ | ALL linkage statistics |
| MIX | MOD |

Legend

Notation: StatisticAMOI(MOI). Dominant (D), additive (A), recessive(R).

Colors correspond to the colored numbers in Additional file 1: Table S1.
